# Crowdsourced Data for Physical Activity-Built Environment Research: Applying Strava Data in Chengdu, China

**DOI:** 10.3389/fpubh.2022.883177

**Published:** 2022-04-29

**Authors:** Linchuan Yang, Bingjie Yu, Pengpeng Liang, Xianglong Tang, Ji Li

**Affiliations:** School of Architecture, Southwest Jiaotong University, Chengdu, China

**Keywords:** physical environment, cycling, running, spatial inequality, Strava, health, physical activity, Chengdu

## Abstract

The lack of physical activity has become a rigorous challenge for many countries, and the relationship between physical activity and the built environment has become a hot research topic in recent decades. This study uses the Strava Heatmap (novel crowdsourced data) to extract the distribution of cycling and running tracks in central Chengdu in December 2021 (during the COVID-19 pandemic) and develops spatial regression models for numerous 500 × 500 m grids (*N* = 2,788) to assess the impacts of the built environment on the cycling and running intensity indices. The findings are summarized as follows. First, land-use mix has insignificant effects on the physical activity of residents, which largely contrasts with the evidence gathered from previous studies. Second, road density, water area, green space area, number of stadiums, and number of enterprises significantly facilitate cycling and running. Third, river line length and the light index have positive associations with running but not with cycling. Fourth, housing price is positively correlated with cycling and running. Fifth, schools seem to discourage these two types of physical activities during the COVID-19 pandemic. This study provides practical implications (e.g., green space planning and public space management) for urban planners, practitioners, and policymakers.

## Introduction

Physical activity is normally defined as “any bodily movement produced by skeletal muscles that results in energy expenditure” (p. 126) (1). It includes walking, running, cycling, mountain climbing, and Tai Chi. Ample evidence reveals that physical activity decreases the incidence rate of chronic and non-communicable diseases, which have become urgent urban public health problems. Physical activity can also help individuals reduce their risk of developing cardiovascular disease, obesity, and many metabolic syndromes (2). Apart from improving physical health, physical activity plays an important role in alleviating depression, cognitive impairments, anxiety, neurosis, and stress. However, physical inactivity (or the insufficiency of physical activity) has plagued many countries worldwide for various reasons, including the deterioration of the urban environment and the sedentary lifestyles of residents. Therefore, promoting the physical activity of residents is being widely advocated worldwide. Even top academic journals *Nature* and *The Lancet* have published a few papers on physical activity (2–6).

The built environment means the physical attributes of the urban landscape (7–10). Many studies have established significant associations between the neighborhood-level built environment (e.g., density, diversity, design, and accessibility) and the physical activity of residents. Various studies have also proven that urban design (e.g., green space, street environment, and service facilities) stimulates residents to engage in physical activities, thereby improving their health (11). Therefore, identifying the association between the built environment and the physical activity of residents is critical in guiding healthy urban planning and creating a livable community environment.

As fundamental forms of physical activities, walking, running, and cycling are extensively carried out in public spaces (i.e., parks, streets, and playgrounds). These activities are suitable for all age groups because pedestrians, runners, and cyclers can adjust their activity intensity at will according to their physical conditions. As effective ways of eliminating the sedentary living habits of individuals, these activities are also typified by a low risk of injury. Therefore, they have recently attracted much attention from residents and academia alike (12–18).

Against this backdrop, this study takes central Chengdu (China) as the study area and scrutinizes the relationship between the built environment and the physical activity (including cycling and running) of residents. First, crowdsourced data has the potential to help researchers solve complicated but meaningful questions and gain new and thought-provoking findings. In stark contrast to previous studies that mostly use “small” physical activity data, we use secondary data from the Strava Heatmap (novel crowdsourced data) in December 2021 to examine the distribution of cycling and running tracks in central Chengdu. We then assess diverse types of built environmental indicators based on multi-source big data and the “5Ds” model. We also develop spatial regression models to analyze the relationship between the built environment and physical activity. Notably, we focus on the different impacts of the built environment on cycling and running and offer practical implications (e.g., green space planning and public space management) for urban planners, practitioners, and policymakers.

The contributions of this study can be summarized as follows. First, most physical activity studies have used small data, such as questionnaire surveys (which often cover hundreds of people), whereas this study uses the Strava Heatmap to obtain the spatio-temporal distribution of physical activity tracks (aggregate data) in Chengdu. Secondly, previous research on the impact of the built environment on cycling/running mostly focuses on a single mode; that is, only very few studies have compared the impact of the built environment on cycling and running. Third, this study integrates new built-environment indicators. Specifically, the night is a vital period for residents to cycle or run. However, the light index has rarely been considered. Furthermore, green and blue space indicators (e.g., river line length) and economic variables (e.g., housing price) have seldom been discussed in the literature.

The remainder of this study proceeds as follows. Section “Literature Review” presents a review of studies on the impact of the built environment on the physical activity of residents. Section “Data” describes the basic overview of the study area and introduces the data and variables. Section “Methodology” presents the research methods. Section “Results” reveals the modeling results. Section “Discussion” provides a critical discussion of the outcomes. Section “Conclusion” summarizes the major conclusions, policy implications, and research limitations.

## Literature Review

The correlation between the built environment and activity behavior has always been a hot research topic. Evaluating the built environment is fundamental to this research field. The most popular framework for assessing the built environment is the “3Ds” (or “5Ds” and “7Ds”) model, which was seminally proposed by Cervero and Kockelman (7) in 1997. The “3Ds” model suggests that the built environment is assessed by density, diversity, and design. Over 10 years later, Ewing and Cervero (19) expanded the “3Ds” model to the “5Ds” model (adding destination accessibility and distance to transit) and the “7Ds” model (adding demand management and demographics).

Numerous studies have assessed the built environment using the “3Ds” or “5Ds” model and its relationship with activity behavior using econometric methods (20). First, some scholars point out that a high residential density contributes to extending the walking time of residents (21). However, a few scholars observe that residents in high-density areas have a low willingness to walk because these areas, which are generally characterized by a high flow of people and vehicles, are likely to incur traffic accidents. Second, Li et al. (22) confirmed that areas with mixed land use provide residents with various travel destinations and service facilities (e.g., parks, schools, retail, public transportation, and entertainment). Therefore, mixed land use facilitates “transport walking” (e.g., walking to school) (23) and “leisure walking” (e.g., walking to the park) and fosters the enthusiasm of residents for walking. Third, Yang et al. (24) developed multilevel models to examine the correlation between intersection density, a typical design element, and the travel propensity of older adults in Hong Kong.

Many scholars have recently explored the impact of the built environment on walking from the micro perspective of urban design. For example, ample evidence supports significant correlations between infrastructure quality (e.g., sidewalk quality and lighting facilities) and walking behavior (25–27). Boarnet et al. (28) argued that pedestrian crossings, traffic signals, overpasses, and street facilities have a strong effect on walking, whereas natural elements and architectural features only have a marginal impact. Lamíquiz et al. (29) investigated the influence of Madrid's urban form on walking behavior based on a spatial syntax and found that walking frequency is negatively correlated with the route length of the street but is positively related to spatial intelligibility. Interestingly, pedestrians are likely to avoid perceived long routes. Based on a large-scale official travel survey and Google Street View imagery data, Yang et al. (30) concluded that the eye-level street greenery has a positive effect on the walking time of older adults in Hong Kong, but the effect varies across space (spatially heterogeneous effect). Using the random forest modeling approach, Yang et al. (31) suggested that the eye-level street greenery has a non-linear effect on older adults' walking propensity in Hong Kong.

The impacts of the built environment on cycling have also been extensively studied. Heinen (32) highlighted a positive correlation between population density and cycling trip rate. Guo and He (33) revealed that population density is unassociated with to-metro bike-sharing use. Tu et al. (34) concluded that cycling trip frequency is positively correlated with the proportion of residential land and green space area, floor area ratio (FAR), and road density but is negatively related to intersection density. Wang and Chen (35) found that certain indicators, such as land-use mix and access to commercial service facilities, are positively associated with cycling. Kaltenbrunner et al. (36) carried out a cycling study in Barcelona and revealed that people's demand for cycling increases when they enter the city from the edge. Many scholars agree that supporting facilities, such as bicycle lanes and stations, parking spaces, signal lights, and drinking facilities, have a discernable impact on cycling (37, 38).

## Data

### Study Area

As the capital of Sichuan Province (China), Chengdu is one of the national central cities as well as core cities in West China. Chengdu is located in the west of the Sichuan Basin and has a subtropical monsoon humid climate. Besides, Chengdu enjoys the honor of a “Park City,” a national forest city, as well as a national civilized city. As of the end of 2020, the permanent resident population of Chengdu was 20.94 million, according to the data of the Seventh National Census. The GDP reached 1,771.67 billion yuan.

**Figure 1:**
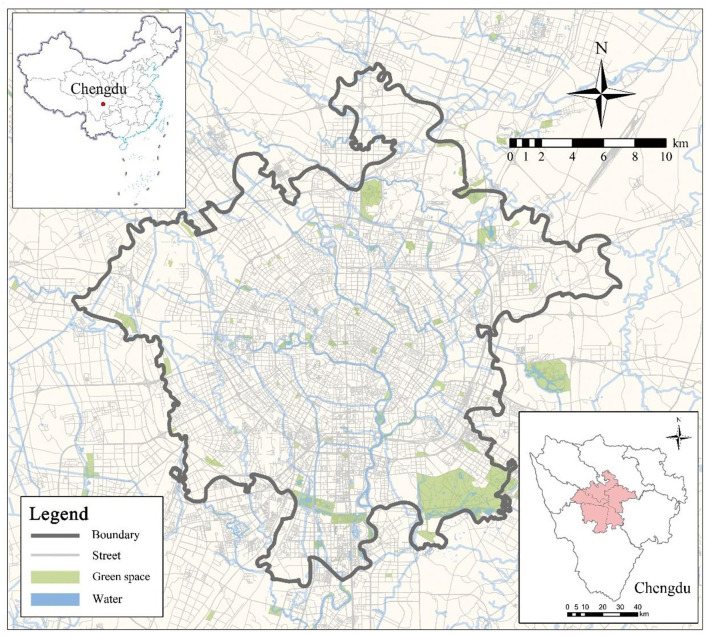
The study area: central Chengdu.

This study selects five districts in central Chengdu (*wu chengqu*; namely Jinniu, Qingyang, Jinjiang, Chenghua, and Wuhou districts) as the study area (Figure 1).

### Strava Data

Strava is one of the largest sports/exercise apps in the world, and currently, it has more than 50 million users worldwide. This app allows users to track their cycling, running, water, and winter activities and has an interactive map display interface to present the Strava Heatmap of all its users across the globe. Notably, walking data is not recorded in Strava.

In 2021, Strava had 1.8 billion physical activity records, representing an annual increase of 38%. The Strava Heatmap not only provides road information and preferred routes for runners and cyclers but also offers the physical activity track distribution with a monthly update.

**Figure 2:**
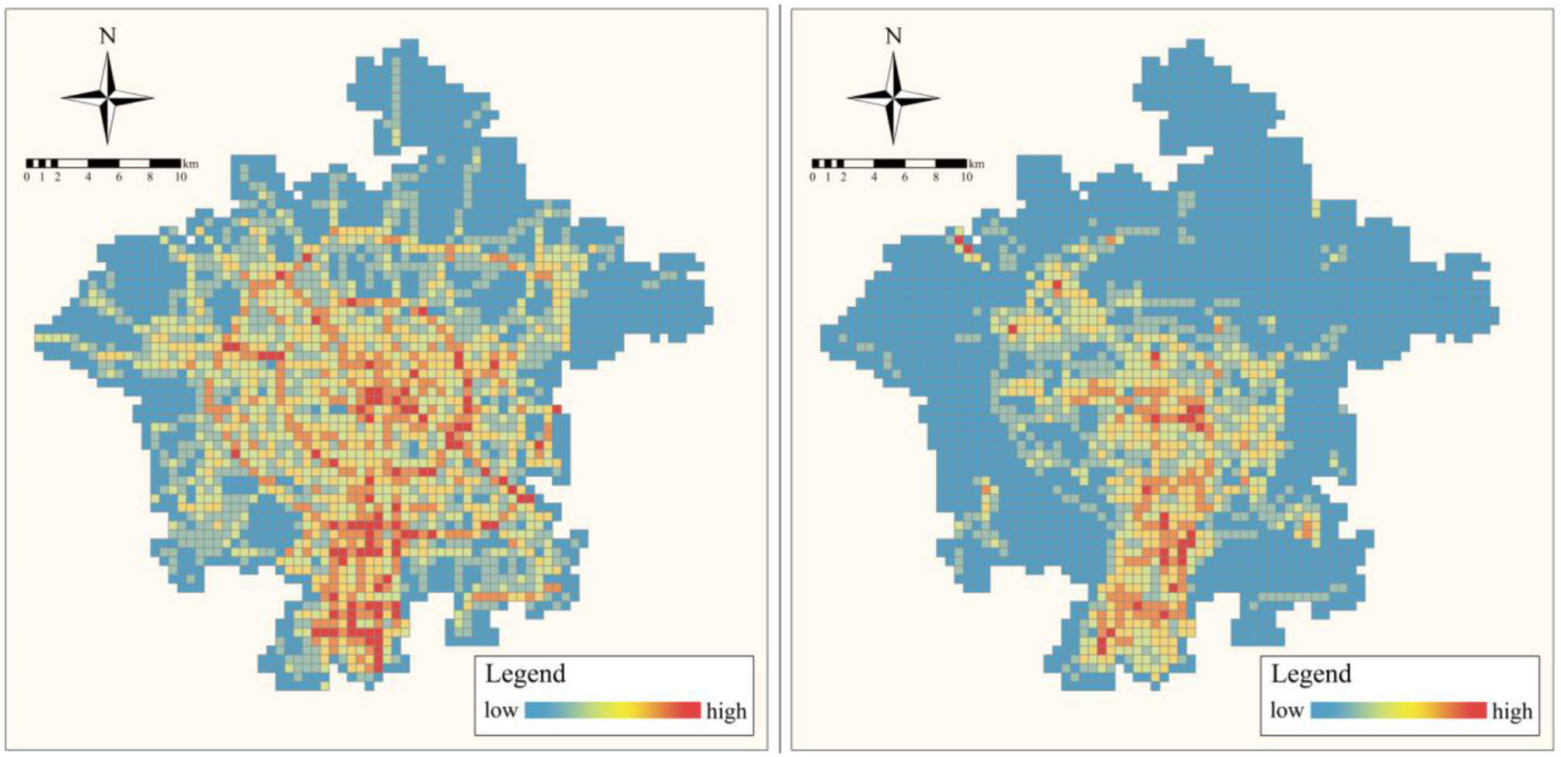
The spatial distribution of the two indices. **(A)** Cycling index. **(B)** Running index.

We collected the distribution map of cycling and running tracks in Chengdu in December 2021 from the official website of Strava (https://www.strava.com/heatmap) and obtained the cycling and running heat data. Afterward, we divided our study area into 500 × 500 m grids through the grid processing feature in GIS. We obtained the cycling and running intensity/index in each grid. The spatial distribution of the two indices is shown in Figure 2.

### Built Environment Data

As a new data source, POI (point of interest) data can reflect the spatial layout characteristics of various service facilities. We used Python to crawl POI data in Chengdu from the Gaode map (https://www.amap.com/) and to obtain information on number of residential buildings, enterprises, schools, and stadiums in the 500 × 500 m grid. We calculated the land-use mix degree using 22 POI categories.

We obtained building outline, road, and traditional bus transit (abbreviated to “bus” hereafter) and metro network data of Chengdu from Gaode Map and calculated the FAR, road density, and number of bus and metro stations in each grid using GIS.

We gathered water and green space data from the Geographic Information Service Center of the State Bureau of Surveying and Mapping. Using the data, we calculated water area, river line length, and green space area in each grid.

We collected housing price data from the website of Lianjia (https://cd.lianjia.com) and retrieved lighting data with a resolution of 500 × 500 m from the website of the US National Oceanic and Atmospheric Administration(https://ngdc.noaa.gov).

### Variables

This study chooses cycling and running, two types of physical activities, as the dependent variables. The selection of the independent variable follows the “5D_S_” model. Moreover, this study adds housing price, an economic attribute, as an independent variable. The summary of the dependent and independent variables is shown in Table 1.

**Table 1:** Descriptive statistics of the variables.

**Variables**		**Std**	**Mean**	**Min**	**Max**
**Dependent variables**
Cycling index		28.55	27.49	0	151
Running index		23.22	13.32	0	160
**Independent variables**
Density	Residential building density (1/km^2^)	33.84	21.00	0	272
	FAR	1.04	1.19	0	7.01
Diversity	Land-use mix	0.23	0.56	0	0.84
Design	Water area (ha)	1.22	0.34	0	15.35
	River line length (km)	0.27	0.17	0	1.43
	Green space area (ha)	3.26	0.86	0	25
	Light index	21.36	87.43	30	186
	Road density (km/km^2^)	2.72	3.85	0	14.71
Distance to transit	Number of bus and metro stations	1.33	1.17	0	8
Destination accessibility	Number of enterprises	28.94	12.88	0	460
	Number of schools	2.14	0.88	0	41
	Number of stadiums	1.65	0.71	0	20
Economic attribute	Housing price (10^4^ yuan/m^2^)	0.35	1.38	0.64	3.83

## Methodology

We adopted a linear regression model (i.e., OLS model) as our basic model, given that our two dependent variables are continuous. However, the presence of spatial autocorrelation (or spatial dependence) violates the traditional OLS regression assumptions. As such, spatial econometric or spatial regression models, such as the spatial lag model (SLM) and spatial error model (SEM), need to be applied to address the spatial autocorrelation and to improve the reliability of our findings and conclusions. Indeed, the application of spatial econometric models is becoming increasingly popular in built environment research, highlighting the obvious shortcomings of traditional OLS regression methods (the inability to address the spatial autocorrelation). Prior to the use of spatial regression models, Moran's *I* (or Geary's *C*, Getis'*G*) statistics often need to be calculated to detect the presence/absence of spatial autocorrelation (39).

Two basic spatial regression models—the SLM and SEM—are often used in prior studies. The SLM specifically handles the spatial autocorrelation in the dependent variable by adding a spatially lagged dependent variable into the linear regression model. It is expressed as follows.


$$\begin{array}{l} Y=\rho WY+X\beta +\varepsilon , \end{array}$$

where *Y* is the dependent variable (continuous variable); *W* is a spatial weight matrix; *WY* denotes the spatially lagged dependent variable; *X* is the independent variable; β is the coefficient of *X*; ρ is the spatial autocorrelation parameter; and ε is spatially uncorrelated residuals. If ρ = 0, then the SLM is equivalent to the OLS model.

The SEM specifically addresses the spatial autocorrelation in the residual. It is expressed as follows.


$$\begin{array}{l} Y=X\beta +\varepsilon ,\\ \varepsilon =\lambda W\varepsilon +u, \end{array}$$

where ε is spatially correlated residuals; *u* is spatially uncorrelated residuals; λ is the spatial autocorrelation parameter; and other parameters are as defined above. If λ = 0, then ε becomes spatially uncorrelated residuals, and the SLM is equivalent to the OLS model.

Unlike the SEM, the estimated coefficients of the SLM cannot be directedly interpreted, and they only describe the directed effects of the independent variables instead of indirect effects or total effects.

## Results

**Figure 3:**
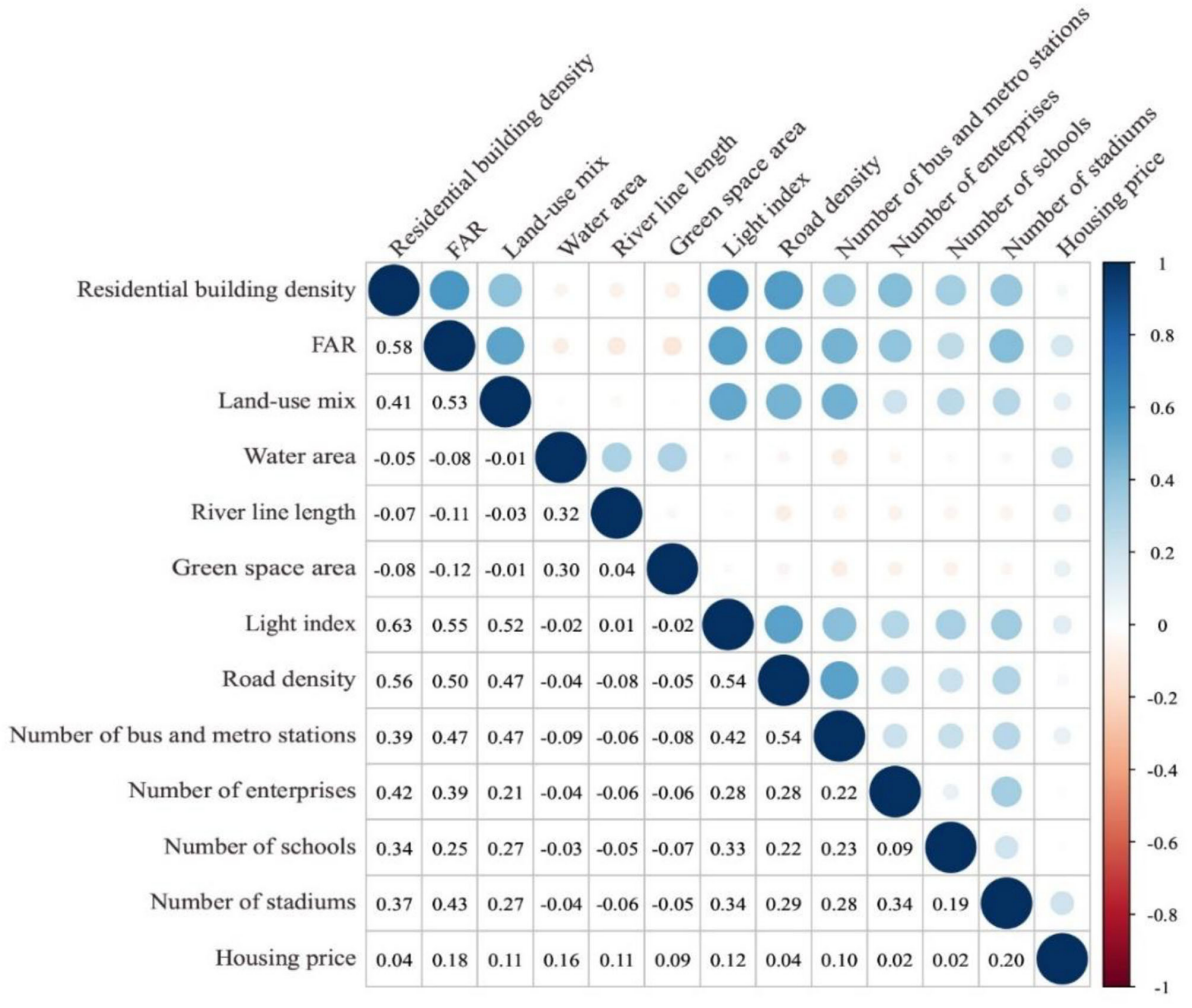
Pair-wise correlation analysis result.

A pair-wise correlation analysis is conducted to detect the multicollinearity between the independent variables before regression. Figure 3 shows the outcome, which indicates no multicollinearity in the data (correlation <0.7).

We initially estimated the non-spatial regression models, that is, the OLS models, and present the results in Table 2. We find that most independent variables are significant at the 5% level, which agrees with our expectations.

**Table 2:** OLS modeling results.

**Variables**	**Cycling index**		**Running index**	
	**Coef**.	**Std. Err**.	**Coef**.	**Std. Err**.
Residential building density	0.097	0.070	0.106	0.059
FAR	0.270	0.552	1.735^**^	0.468
Land-use mix	6.695^**^	2.232	−2.983	1.892
Water area	2.280^**^	0.361	3.896^**^	0.306
River line length	−0.373	1.585	3.965^**^	1.344
Green space area	0.610^**^	0.129	0.831^**^	0.110
Light index	0.200^**^	0.027	0.173^**^	0.023
Road density	2.785^**^	0.201	1.415^**^	0.170
Number of bus and metro stations	3.909^**^	0.377	0.749^*^	0.32
Number of enterprises	0.122^**^	0.016	0.044	0.013
Number of schools	−0.590^**^	0.202	−0.099	0.171
Number of stadiums	1.434^**^	0.278	1.396^**^	0.236
Housing price	14.392^**^	1.195	20.438^**^	1.013
Constant	−33.056^**^	2.475	−41.418^**^	2.098
**Performance statistics**
*F*-statistic	187.46		155.43	
*p*-value	<0.001		<0.001	
*R*-squared	0.468		0.421	
Adjusted *R*-squared	0.465		0.419	
Number of observations	2,788			

** *Significant at the 1% level.*

* *Significant at the 5% level*.

**Figure 4:**
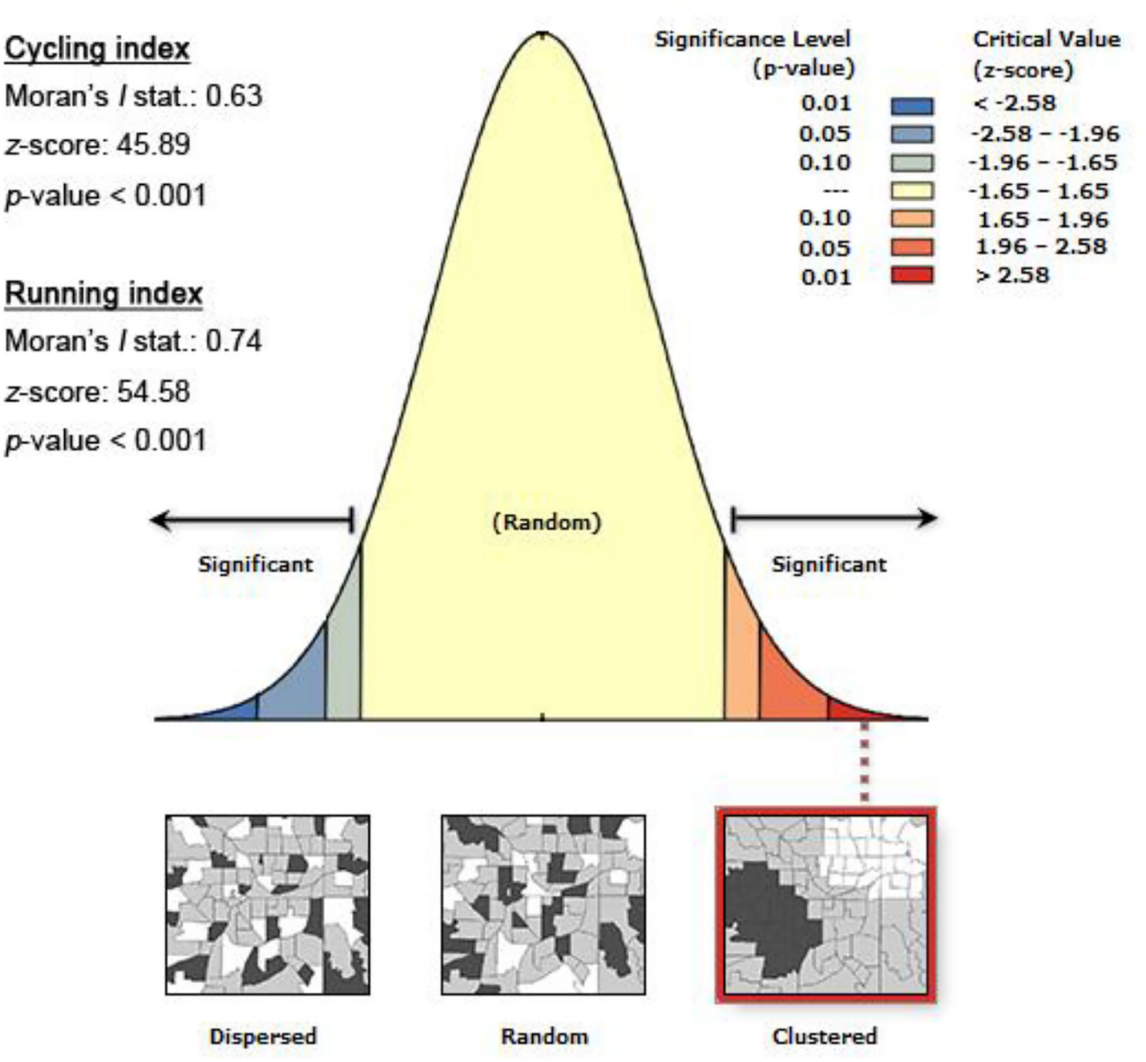
Moran's *I* statistics for the cycling and running indices.

We calculated the Moran's *I* statistics for the cycling (0.63) and running (0.74) indices in ArcGIS and presented the calculation results in Figure 4. Both statistics are significant at the 1% level, indicating the presence of a positive spatial autocorrelation. A z-score of 45.89 or 54.58 suggests a <1% likelihood for this clustered pattern to be the result of random chance. In sum, using spatial regression models is critical.

Table 3 presents the spatial modeling results. The SLM slightly outperforms the SEM in assessing the two indices, as indicated by its lower AIC and higher log-likelihood. Therefore, we used the SLM results for the subsequent interpretation. Moreover, the spatial autocorrelation parameter is positive and significantly different from zero (*p*-value < 0.01), which indicates the presence of a positive spatial autocorrelation and echoes the Moran's *I* statistic. This observation agrees with our expectations.

**Table 3:** Spatial modeling results.

**Variables**	**Cycling index**				**Running index**			
	**SLM**		**SEM**		**SLM**		**SEM**	
	**Coefficient**	**Std. Err**.	**Coefficient**	**Std. Err**.	**Coefficient**	**Std. Err**.	**Coefficient**	**Std. Err**.
Residential building density	−0.033	0.060	−0.054	0.082	−0.124^**^	0.041	−0.274^**^	0.058
FAR	−1.468^**^	0.475	−1.465^**^	0.551	−0.084	0.327	−0.012	0.379
Land-use mix	3.805^*^	1.920	5.524^*^	2.253	−0.940	1.317	0.077	1.542
Water area	1.119^**^	0.311	0.914^**^	0.354	1.966^**^	0.217	2.359^**^	0.241
River line length	1.249	1.361	1.667	1.550	4.777^**^	0.936	7.155^**^	1.057
Green space area	0.295^**^	0.111	0.307^*^	0.140	0.422^**^	0.077	0.625^**^	0.098
Light index	0.032	0.024	0.333^**^	0.049	0.044^**^	0.016	0.241^**^	0.050
Road density	1.992^**^	0.177	2.646^**^	0.205	0.829^**^	0.120	1.039^**^	0.141
Number of bus and metro stations	3.008^**^	0.325	2.764^**^	0.333	0.308	0.223	0.148	0.223
Number of enterprises	0.089^**^	0.014	0.099^**^	0.016	0.025^**^	0.009	0.037^**^	0.011
Number of schools	−0.549^**^	0.173	−0.643^**^	0.185	−0.210^**^	0.119	−0.350^**^	0.125
Number of stadiums	0.864^**^	0.239	0.556^**^	0.245	0.717^**^	0.164	0.496^**^	0.164
Housing price	4.336^**^	1.049	1.840	1.496	4.172^**^	0.742	3.266^**^	1.073
Constant	−11.470^**^	2.225	−20.856^**^	4.613	−11.492^**^	1.576	−18.359^**^	4.877
p\λ	0.601^**^	0.020	0.677^**^	0.020	0.783^**^	0.014	0.851^**^	0.013
**Performance statistics**
*R*-squared	0.605		0.603		0.719		0.727	
Log likelihood	−12,089.0		−12,125.7		−11,124.3		−11,128.1	
AIC (Akaike information criterion)	24,208.1		24,279.4		22,278.6		22,284.2	
Number of observations	2,788							

** *Significant at the 1% level.*

* *Significant at the 5% level*.

The cycling index modeling result demonstrates that the “5Ds” variables, namely density, diversity, design, distance to transit (or transit accessibility), and destination accessibility, are significantly related to the cycling index in the study area. Specifically, the cycling index is positively correlated with land-use mix, water area, green space area, road density, number of bus and metro stations, number of enterprises, and number of stadiums (*p*-value < 0.05) but is negatively associated with FAR and number of schools (*p*-value < 0.01). Interestingly, residential building density, river line length, and the light index insignificantly affect the cycling index, and housing price is positively related to this index (*p*-value < 0.01).

The modeling results for the running index show that this index is positively associated with water area, river line length, green space area, the light index, road density, number of enterprises, and number of stadiums (*p*-value < 0.01) but is negatively correlated with residential building density and number of schools (*p*-value < 0.01). Nevertheless, FAR, land-use mix, and number of bus and metro stations are insignificant at the 5% level. Furthermore, housing price is positively connected to the running index (*p*-value < 0.01).

The above outcomes suggest that the impacts of some built environmental indicators on cycling and running are highly consistent (e.g., water area, green space area, and road density), whereas those of some other attributes (e.g., residential building density, FAR, river line length, and number of bus and metro stations) vary considerably. Most of our findings align with those of previous studies. However, our findings related to FAR, number of schools, and river line length substantially differ from those of the literature.

We compare the magnitude of the coefficients in the OLS and spatial econometric models and find that the effects of most independent variables on the dependent variables are magnified by the presence of a positive adjacency effect. In other words, these coefficients are usually bigger in the OLS models than in the spatial econometric models because of the feedback from the spatially lagged dependent variable (addressed by the SLM) or spatially auto-correlated residuals (addressed by the SEM).

## Discussion

### Density

Residential building density and FAR have either negative or insignificant effects on the cycling and running indices, which contradicts the results of previous studies. Unlike cities in North America, Europe, and Oceania, where walkable neighborhoods/areas are a rarity, Chengdu is a high-density city with constant urban expansion. Therefore, the impact of residential building density and FAR in Chengdu on physical activity may differ from that observed in western countries. The contrasting results may also be ascribed to the habits of Chinese residents, who mostly prefer to use the Strava app to record their leisure physical activities. Compared with their counterparts in low-density areas, Chinese residents tend to engage in physical activities in public spaces marked by clear vision, low traffic flow, and high safety. Some evidence also suggests that density plays a vital role in transportation walking, and its correlation with leisure walking (more broadly, leisure physical activity) remains unclear (40).

Areas with high residential building density and FAR may be unfriendly to running and cycling activities because of heavy pedestrian and traffic flows, poor safety, and poor exercise experience. In addition, high population density and road density tend to induce traffic accidents.

### Diversity

Principals of New Urbanism assert that mixed land use promotes the active travel (an essential component of physical activity) behavior of residents. Interestingly, in contrast to classical wisdom, we find no significant relationship between land-use mix and cycling/running, possibly because areas with mixed land-use have abundant facilities, thereby allowing residents to achieve various travel purposes within a short distance and subsequently reducing their physical activity. Moreover, we argue that mixed land use does not affect physical activity in only one direction. Specifically, mixed land use not only provides residents with abundant facilities and various travel destinations but also makes this area noisy and crowded, thereby hindering their physical activity (41).

### Design

Water and green space areas are positively correlated with cycling and running, indicating that these areas can promote these two types of physical activities. In other words, in grids with a larger water area and green space area, the cycling and running indices are higher. Previous studies show that urban green space area plays an essential role in facilitating the physical activity of residents and enhancing their health (42). Moreover, urban green space provides residents with a wonderful visual and psychological experience by supplying them with a pleasant natural landscape. Therefore, urban green space significantly increases the physical activity frequency and duration of residents. Similarly, Gong et al. (43) confirmed that elderly men living in communities with more green space regularly engage in more physical activities than those residing in communities with limited green space. Cohen et al. (44) found that the physical activity of women is significantly correlated with number of parks located within a mile of their residence.

River line length shows a positive connection with running but not with cycling, thereby suggesting that the adjacency to rivers stimulates running behavior. Runners attach great importance to the environmental quality of their routes. By contrast, cyclers generally focus on the natural scenery in a broader range, thereby driving their preference for spacious and continuous greenways. Such a difference explains why the government is more inclined to build running lanes rather than dedicated cycling lanes around the waterfront. In sum, runners are more hydrophilic than cyclers.

The light index has a positive association with running but not with cycling. Most city dwellers in China are restricted to the “9 a.m. to 5 p.m.” (*zhao jiu wan wu*) working schedule (go to the office at 9 a.m. and get off work at 5 p.m.). Therefore, the night is a popular time for these residents to engage in physical activities. Night running has become the main exercise model for these residents. Those areas with a high light index provide clear spatial visibility, thereby improving the sense of security of runners. This argument is supported by Painter (45), who believed that a dark environment inhibits the physical activity of residents. However, cyclers, who tend to perform outdoor activities during the daytime, are hardly affected by the light index.

Road density is positively correlated with cycling and running. The transportation network of dense-road areas has good connectivity, which is conducive to traffic micro-circulation (*wei xunhuan*), and thus encourages many kinds of physical activities. Moreover, continuous footpaths, small regions, and dense intersections can slow down the speed of vehicles and provide residents with a safe walking/running/cycling (active travel) environment.

### Distance to Transit (Transit Accessibility)

Given its potential to relieve various contemporary urban problems such as traffic congestion and environmental degradation, transit has received substantial attention from policymakers, planners, and scholars in recent years. Countries across the world have also started to advocate and promote the use of public transit. Moreover, we feel that international readers may be unfamiliar with the characteristics of buses in our study area, so we introduce them here. In Chengdu and other Chinese cities, buses, which run in mixed traffic on an army of overlapping lines with different headways, are considered the pervasive transit mode because of their dense and extensive networks, great temporal and geographical coverage, and high frequency (e.g., headway = 3 min), especially in the central city.

We find that transit accessibility (described by number of bus and metro stations in this study) has a significant correlation with cycling but not with running. This finding may be ascribed to the fact that Chinese residents prefer to choose public spaces (i.e., community parks, stadiums, and squares) with a low correlation with transit for their running activities, especially leisure running activities. Moreover, those areas with abundant transit stations (high transit accessibility) generally have heavy traffic and noisy environment and are thus unsuitable for leisure physical activities.

Distance to transit, one of the “5Ds” variables, should not be narrowly understood (just as a “distance”) in “activity behavior-built environment” studies, especially in those focusing on transit use-related activity behavior (e.g., transit ridership and the first/last mile travel). We suggest that it should be interpreted as “transit accessibility.” As some transport literature (46–48) suggests, apart from “to-transit accessibility” (or accessibility to transit), transit accessibility has an additional far-reaching meaning, namely “by-transit accessibility” (or accessibility by transit). By-transit accessibility is much less well-known than to-transit accessibility, which refers to the ease of accessing transit.

When the transit service is pervasive but poorly designed and planned such that people have good to-transit accessibility but poor by-transit accessibility, they still cannot satisfactorily use the service to travel. As such, by-transit accessibility is also essential, although it is always ignored. It has profound implications for travel/activity behavior (46, 47).

To-transit accessibility is often measured by (transit station) “location” variables, including travel distance or time to nearby transit stations (the first/last mile). By-transit accessibility is commonly measured by (transit) “service” variables, such as transit travel time to the city center and transit frequency.

### Destination Accessibility

Schools and stadiums are often regarded as significant places for promoting the physical activity of residents. Schools, which are characterized by small internal traffic flow and high safety, provide excellent venues for many kinds of physical activities. People can participate in physical activities within campus grounds (e.g., street, playground, green space, and public space) without any worries for their safety. Nonetheless, we find that school accessibility is negatively correlated with the cycling and running behavior of residents, which can be ascribed to the school management policy during the COVID-19 pandemic in China. Indeed, campuses in China have become semi-open or fully closed since the COVID-19 outbreak, thereby preventing nearby residents from visiting. Therefore, physical activity in schools has decreased significantly during the pandemic.

### Housing Price

Housing price has positive associations with running and cycling (*p*-value < 0.01), indicating that areas with higher housing prices have greater physical activity intensity.

Many urban development projects (e.g., environmental remediation, brownfield site redevelopment, and green infrastructure construction) can introduce new amenities, improve the living quality of nearby residents, and subsequently increase the value of nearby land or housing. An increase in housing value generates a “squeeze” effect on low-income residents or people with low housing affordability (especially housing renters, not housing owners) and may compel them to move out of their areas. Meanwhile, improvements in living quality can attract high-income groups to move in and occupy high-quality spaces, thereby inducing “environmental gentrification.” Although these amenities are either fully open (e.g., riverside park and citizen square) or semi-open (e.g., high-end community and lakeside villa), spatial inequality remains. Environmental improvement may exacerbate spatial inequality and undermine environmental justice.

### Public Goods and Services as the Nature of the City: A Theory

In this study, several public services—including transit, school, and stadium—are the correlates of physical activity indices. This finding reminds the authors of an interesting and thought-provoking theory proposed by a famous Chinese urban planning scholar at Xiamen University, Yanjing Zhao. The related paper was published in a top Chinese planning journal, *City Planning Review*, in 2009. We decide to discuss this captivating theory here with the aim of spreading it to international academia.

What defines a city? What distinguishes a city from a town (or a village, a hamlet)? What is the nature of the city? The answers are not as clear-cut as they seem to be. Organizations and scholars have developed numerous city definitions based on different perspectives (e.g., population and the economy). No universal city definition exists. A well-known definition of the city is a large and permanent human settlement, while others include a place with high population density or concentrated economic activities. Generally, the criteria for distinguishing a city from a rural area include, but are not limited to, population size/density, number of dwellings, functionality, non-agricultural occupation (or economic structure), political status, social organization, the symbol of wealth, lifestyle and landscape, and the presence of literate elites. Furthermore, various city categories based on prominent characteristics exist, such as resource-oriented, trading, industrial, political, defensive, and tourism cities.

Zhao (49) explores the nature of the city from the perspective of the institution and innovatively proposes the institutional archetype of the city. He uses his theory to answer a host of questions that traditional urban theories cannot adequately explain (e.g., quality of urbanization). He argues that a city is essentially a place to produce and consume public goods and services (PGSs) (*gonggong chanpin he fuwu*), such as defense security and school, and puts forward that a city “starts from the space where the public services are provided” (p. 9). He points out that what discriminates a city from a rural area is whether or not a transaction of PGSs occurs (e.g., by means of the tax). He then demonstrates the significant role of government or pseudo-governments (e.g., owners committee) in providing PGSs.

Note that Zhao (49) holds that PGSs must be charged through “space.” The perspective is different from that in economics. Under the framework of Zhao (49), the significance of PGSs to a city has been further emphasized compared with others, such as human settlement, economic activities, and population density.

## Conclusion

This study examines the correlation of the built environment with running and cycling, which are measured by using crowdsourced data, that is, the Strava Heatmap. In doing so, this study enhances our understanding of the different impacts of the built environment on the cycling and running activities of residents. The results can guide policymakers and urban planners/designers in formulating the appropriate and effective physical activity intervention measures, promoting healthy urban planning, and creating a livable community-level built environment.

Unlike previous studies that focus on many low-density cities in North America, Europe, and Oceania, this Chengdu-based study does not show that high density and high land-use mix are conducive to physical activities (cycling and running), which may be explained by the strict urban management in China during the COVID-19 pandemic. In line with our expectations and the findings of previous research, we find that water area, green space area, road density, number of stadiums, and number of enterprises significantly promote cycling and running. Nonetheless, river line length and the light index have a significantly positive effect on running, but not on cycling. Interestingly, housing price is positively correlated with cycling and running, whereas number of schools is negatively correlated with the two physical activities, probably due to the strict regulation of schools during the COVID-19 pandemic (prohibiting the entry of city residents).

This study provides some practical implications. First, the negative impact of “environmental gentrification” can be alleviated by adjusting the housing supply. In other words, a fair and shared public space should be created. One approach toward this objective is to offer compound residential land (e.g., commercial housing, talent apartments, and affordable housing), which can reduce the impact of urban green infrastructure renewal on low-income earners. Second, we find that green and blue spaces (i.e., water area, river line length, and green space area) are particularly attractive for people engaging in physical activities. In its current plans to construct a “Park City,” Chengdu should set up walking- and cycling-friendly greenways and provide its residents with sufficient green space (e.g., along the main river or on both sides of the road). Third, high-density communities can build a sophisticated slow-moving system and develop a small-block, dense-road model to reduce the adverse impacts of dense traffic flow on runners and cyclists and ensure their safety. Last, public spaces with high physical activity demand should be given sufficient lighting to satisfy the physical activity needs of residents in the evening and at night. This measure may even encourage residents to participate in physical activities in particular time windows.

However, this study also has its limitations. First, due to data unavailability, variables such as bicycle lanes, running tracks, the quality of slow-moving facilities, open/gated community, and weather/climate are excluded from the models (50, 51). Second, this study is grid-based or area-based (aggregate) rather than individual-based (disaggregate). Therefore, great caution is advised when interpreting the modeling results. Making causal and associative inferences about individuals from inferences about the group (grid in this study) to which the individuals belong, which is commonly called the ecological fallacy (also called ecological inference fallacy or population fallacy), should also be avoided (for example, Simpson's Paradox may arise). Individual (micro) correlation cannot be deduced from ecological (macro) correlation. Last, this study only focuses on dealing with spatial autocorrelation. Another distinct characteristic of spatial data is spatial heterogeneity (or spatial non-stationarity), which means that some variables exert larger impacts in some locations but smaller or imperceptible impacts in others. It is overlooked in this study. In sharp contrast with physical processes, social processes are often non-stationary. Different from OLS regression, which uses a single equation to describe global relationships in physical or social processes, the geographically weighted regression (GWR) model creates a multitude of equations for the description and specifically tackles spatial heterogeneity. Therefore, it is a popular approach to modeling social processes. We feel that using the GWR model or its enhanced version—the multiscale GWR model—to scrutinize the spatially varying relationship between physical activity and the built environment is worthy of investigation in further studies.

## Data Availability Statement

The raw data supporting the conclusions of this article will be made available by the authors, without undue reservation.

## Author Contributions

LY: conceptualization, funding acquisition, supervision, and writing—original draft. BY: formal analysis, methodology, and writing—review and editing. PL: formal analysis and writing—review and editing. XT and JL: validation and writing—review and editing. All authors contributed to the article and approved the submitted version.

## Funding

This study was supported by the Key Program of the Center on Child Protection and Development, Sichuan (No. ETBH2021-ZD001), the Sichuan Science and Technology Program (No. 2022JDR0178), the Fundamental Research Funds for the Central Universities of China (No. 2682021CX097), and the National Natural Science Foundation of China (No. U20A20330).

## Conflict of Interest

The authors declare that the research was conducted in the absence of any commercial or financial relationships that could be construed as a potential conflict of interest.

## Publisher's Note

All claims expressed in this article are solely those of the authors and do not necessarily represent those of their affiliated organizations, or those of the publisher, the editors and the reviewers. Any product that may be evaluated in this article, or claim that may be made by its manufacturer, is not guaranteed or endorsed by the publisher.
